# Prenatal diagnosis of Down syndrome combined with transient abnormal myelopoiesis in foetuses with a GATA1 gene variant: two case reports

**DOI:** 10.1186/s13039-023-00658-w

**Published:** 2023-10-19

**Authors:** Hui Tang, Jingjing Hu, Ling Liu, Lijuan Lv, Jian Lu, Jiexia Yang, Jiaqi Lu, Zhenhui Chen, Chaoxiang Yang, Dan Chen, Jintao Fu, Jing Wu

**Affiliations:** 1 Gentic Medical Center, Guangdong Women and Children Hospital, Guangzhou, People’s Republic of China; 2 Laboratory Department, Guangdong Women and Children Hospital, Guangzhou, People’s Republic of China; 3 Radiology Department, Guangdong Women and Children Hospital, Guangzhou, People’s Republic of China; 4 Ultrasound Department, Guangdong Women and Children Hospital, Guangzhou, People’s Republic of China; 5 Pathology Department, Guangdong Women and Children Hospital, Guangzhou, People’s Republic of China

**Keywords:** Transient abnormal myelopoiesis, Down syndrome, Trisomy 21, *GATA1*, Prenatal diagnosis

## Abstract

**Background:**

Down syndrome myeloid hyperplasia includes transient abnormal myelopoiesis (TAM) and the myeloid leukemia associated with Down syndrome (ML-DS). The mutation of *GATA1* gene is essential in the development of Down syndrome combined with TAM or ML-DS. Some patients with TAM are asymptomatic and may also present with severe manifestations such as hepatosplenomegaly and hydrops.

**Case presentation:**

We report two cases of prenatally diagnosed TAM. One case was a rare placental low percentage 21 trisomy mosiacism, resulting in the occurrence of a false negative NIPT. The final diagnosis was made at 36 weeks of gestation when ultrasound revealed significant enlargement of the foetal liver and spleen and an enlarged heart; the foetus eventually died in utero. We detected a placenta with a low percentage (5–8%) of trisomy 21 mosiacism by Copy Number Variation Sequencing (CNV-seq) and Fluorescence in situ hybridization (FISH). In another case, foetal oedema was detected by ultrasound at 31 weeks of gestation. Two foetuses were diagnosed with Down syndrome by chromosomal microarray analysis via umbilical vein puncture and had significantly elevated cord blood leucocyte counts with large numbers of blasts. The *GATA1* Sanger sequencing results suggested the presence of a [NM_002049.4(*GATA1*):c.220G > A (p. Val74Ile)] hemizygous variant and a [NM_002049.4(*GATA1*):c.49dupC(p. Gln17ProfsTer23)] hemizygous variant of the *GATA1* gene in two cases.

**Conclusion:**

It seems highly likely that these two identified mutations are the genetic cause of prenatal TAM in foetuses with Down syndrome.

**Supplementary Information:**

The online version contains supplementary material available at 10.1186/s13039-023-00658-w.

## Introduction

Trisomy 21 is the most common chromosomal abnormality, with a frequency of 1 in 700–800 live births [1]. The myeloid proliferations related to Down syndrome—transient abnormal myelopoiesis and myeloid leukemia—have unique morphologic, immunophenotypic, clinical, and molecular features, including *GATA1* mutation, that justify their separation from other myeloid neoplasms. Myelodysplastic syndrome (MDS) related to Down syndrome is biologically identical to Down-related acute myeloid leukemia (AML); Therefore they are considered as a single entity, ML-DS, in the classification [2, 3].

It is generally accepted that the *GATA1* mutations and trisomy 21 cooperate in causing TAM. Acquired mutations in the hematopoietic transcription factor *GATA1* are found in megakaryoblasts of nearly all individuals together with Down syndrome with TAM and the related acute megakaryoblastic leukemia (DS-AMKL, also called DS-AML M7) [4, 5]. These mutations lead to production of a variant GATA1 protein (GATA1s) that is truncated at its N terminus. Li et al. showed that the dominant action of GATA1s leads to hyperproliferation of a unique, previously unrecognized yolk sac and foetal liver progenitor, and this accounts for the transient nature of TAM and the restriction of DS-AMKL to infants [6]. Approximately 20 to 30% patients with TAM develop Down syndrome AML 1–3 years later after spontaneous regression.

Trisomy 21 is essential for the initiation of TAM, the predominant view is that Down syndrome-associated myeloproliferations result from the loss of control of genes on human chromosome 21, which is estimated to contain 234 protein-coding genes. The encoded molecules belong to several functional classes, such as transcription factors, signalling effectors, epigenetic regulators and miRNAs [7]. Recent studies have shown that when a *GATA1* mutation is introduced into trisomy 21 long-term hematopoietic stem cells (LT-HSCs), it leads to TAM development, where TAM initiation is affected by the overexpression of a subset of chromosome 21 microRNAs (miRNAs) [8].

TAM has a variable clinical presentation. It is usually diagnosed within 8 weeks of birth, early prospective studies demonstrated that a large proportion of patients with TAM are asymptomatic. Only circulating blasts without clinical symptoms, most of them fade in the first three months and do not require treatment [9]. But uncommon clinical features may occur with worsening liver function, increasing organomegaly and leukocytosis, visceral effusions and foetal hydrops, these are often serious manifestations of TAM [2, 10, 11].

Here we present two foetuses, first case was a rare case of confined placental mosaicism resulting in the development of a false negative for NIPT, with foetal TAM manifestation only at 36 weeks of gestation and confirmation of a *GATA1* gene c.220G > A heterozygous variant mutation in trisomy 21. In the second case, foetal hydrops was detected at 31 weeks of gestation, and trisomy 21 was diagnosed by umbilical venipuncture, and the heterozygous variant of the *GATA1* gene c.49dupC was reported for the first time.

## Case presentation

### Case1

**Fig. 1:**
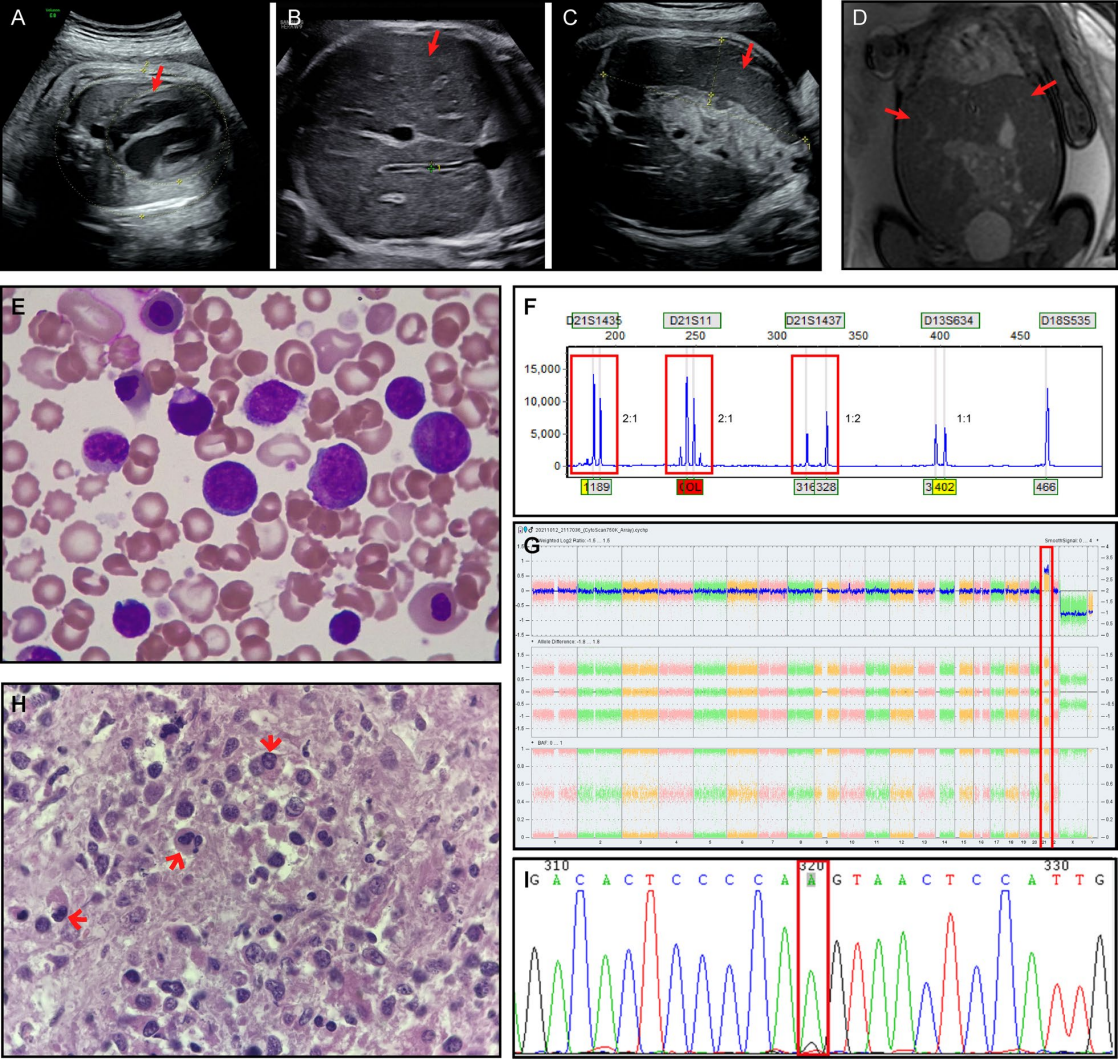
Case1 **A**–**C**: The 36-week ultrasound findings of the foetus. **A** Foetal heart enlargement. **B** The liver of the foetus was enlarged. **C** The foetus had an enlarged spleen. **D** Results of MRI of the foetal abdomen: The foetus had a markedly enlarged liver and spleen. **E** Cord blood film showing blast cells (Case 1) (10 × 100 magnification): The characteristic megakaryoblasts are visible microscopically, the blasts have typical morphology showing fine chromatin with prominent nucleoli and high nuclear cytoplasmic ratio. **F** Electrophoretograms of multiplex QF-PCR amplification with STR markers on chromosomes 18,13 and 21. Electrophoretic profiles observed for trisomy 21 foetus (red boxes). **G** Image from the Affymetrix Chromosome Analysis Suite Software showing trisomy 21. The location of the chromosomal repeat segments is denoted by the red boxes. **H** Histological findings of the foetal liver (Case 1) (Hematoxylin & Eosin staining, 10 × 100 magnification): A large number of intrahepatic structures have been destroyed. Numerous granulocyte (arrow) and blasts are present. Consider this condition to be extramedullary hematopoiesis. **I** Identification of mutations in *GATA1* by Sanger sequencing, as visualized by Chromas software. The base change is indicated by a red boxes. Mutation [NM_002049.4 c.220G > A (p. Val74Ile)] was detected in the foetus

A 35-year-old woman with a 36-week gestation visited the Department of Genetic Medical Center for genetic counselling on the foetal abnormalities revealed by ultrasound examination. This is the first pregnancy. There was no family history of genetic disorders, and the couple was nonconsanguineous. At 13 weeks of gestation, cfDNA testing reported that there were low risks for trisomies 21, 18 and 13. Our scan revealed an enlarged heart, liver, spleen, and splenic vein and portal vein widening, gastric wall thickening, and a small amount of fluid accumulation in the abdominal cavity of foetal. The inferior border of the foetal liver and the inferior border of the spleen both reached the position of the iliac crest. (Fig. 1A–C). The foetus was otherwise normal. For better anatomical evaluation, foetal magnetic resonance imaging (MRI) was performed and showed that the liver and spleen were markedly enlarged (Fig. 1D).

To identify the cause of the foetal hepatosplenomegaly, percutaneous umbilical cord blood was sampled and the cord blood count suggested severe leukocytosis, with a predominance of immature forms, and thrombocytosis were detected by blood cell count. The umbilical cord blood white blood cell (WBC) count was 128.62 × 10^9^/L. Morphological analysis of the umbilical cord blood cells showed a significant increase in the number of white blood cells, a large number of blasts, and naive eosinophils (Fig. 1E). Amniotic fluid was also taken to test for cytomegalovirus and rubella virus. First, the cord blood Quantitative fluorescence polymerase chain reaction (QF-PCR) results appeared on Day 3, suggesting a foetus with trisomy 21(Figs. 1F). However, by this time, intrauterine foetal death had already occurred. Results for amniotic fluid cytomegalovirus and rubella virus were normal. The CMA results suggested that the foetus had trisomy 21 (Fig. 1G).

For further study, liver tissue was taken from the foetus after delivery for pathological examination. Microscopically, the normal liver tissue of the foetus is largely absent. A large number of intrahepatic structures have been destroyed. Numerous granulocyte (arrow) and blasts are present. Consider this condition to be extramedullary hematopoiesis (Figs. 1H). On the other hand, cord blood was Sanger sequenced for the *GATA1* gene, with the aim of exploring the cause of TAM in the foetus. A hemizygote mutation NM_002049.4 c.220G > A (p. Val74Ile) was detected (Fig. 1I). Because we noted that the non-invasive prenatal testing (NIPT) examination of the pregnant woman in early pregnancy suggested a low risk (other hospitals test and raw data is not available). The common cause of false negatives for NIPT is true foetal mosaicism, so we took placental tissue for CNV-seq examination. The centre point of the placenta is a low proportion (6%-8%) of trisomy 21 mosiacism (Additional file 1: Fig. 1). This result was also verified by another method, and FISH analysis detected trisomy 21 signals in 5/100 (5%) uncultured placental cells (Additional file 2: Fig. 2).

### Case2

**Fig. 2:**
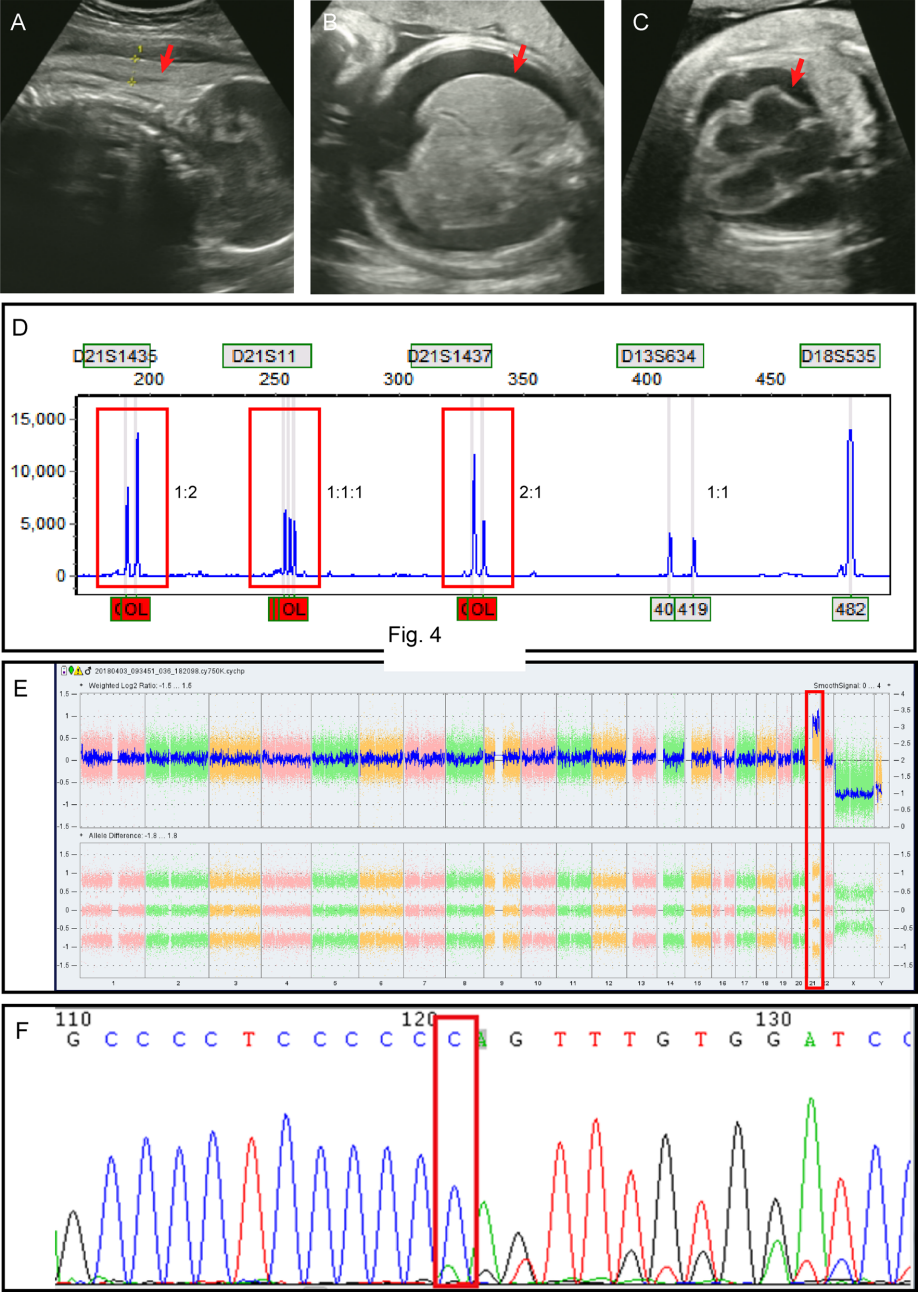
Case2 **A**–**C**: The 31-week ultrasound findings of the foetus. **A** The foetus had thickened skin on the head and chest. **B** Foetal ascites. **C** Foetal heart enlargement combined with pericardial effusion. **D** Electrophoretograms of multiplex QF-PCR amplification with STR markers on chromosomes 18, 13 and 21. Electrophoretic profiles observed for trisomy 21 foetus (red boxes). **E** Image from the Affymetrix Chromosome Analysis Suite Software showing trisomy 21. The location of the chromosomal repeat segments is denoted by the red boxes. **(F)** Identification of mutations in *GATA1* by Sanger sequencing, as visualized by Chromas software. The base change is indicated by a red boxes. Mutation [NM_002049.4 c.49dupC(p. Gln17ProfsTer23)] was detected in the foetus

A 39-year-old pregnant woman at 31 weeks of gestation was referred to our institution due to foetal oedema. The woman is pregnant for the fourth time and has already delivered two daughters, both healthy, missed abortion once. A family history of genetic diseases and consanguineous marriage were denied by the couple. No Down syndrome screening was performed during pregnancy. Ultrasound showed foetal oedema including pericardial effusion and peritoneal effusion, and skin oedema, a biparietal diameter, an abdominal circumference greater than the gestational week, and mild amniotic fluid excess was observed **(**Figs. 2A–C**)**.

To identify the cause of the foetal hydrops, percutaneous umbilical cord blood was sampled and the cord blood count revealed severe leucocytosis, as well as thrombocytopenia. The cord blood leukocyte (WBC) count was 64.32 × 10^9^/L. The details are shown in Table 1. Morphological analysis of the umbilical cord blood cells also showed a significant increase in the number of white blood cells, a large number of blasts, and naive eosinophils. The QF-PCR and CMA results suggested that the foetus had trisomy 21 (Figs. 2D and E). Cord blood was Sanger sequenced for the *GATA1* gene, which is hemizygous mutation NM_002049.4 c.49dupC (p. Gln17ProfsTer23) (Fig. 2F). The patient and her family eventually chose to abandon the pregnancy.

**Table 1:** Foetal blood values

	Case 1	Case 2
WBC	128.62 × 10^9^/L	64.32 × 10^9^/L
Hb	101 g/L	98 g/L
Hct	32.1%	30.9%
Platelet count	416 × 10^9^/L	90 × 10^9^/L
ALT	162 U/L	–
AST	141 U/L	–
Albumin	24.6 g/L	–

## Method

### Ultrasound examination

GE VoIuson E8 color Doppler ultrasound system (GE Medical Systems, Zipf) which equipped with a 2–5 MHz twodimensional convex array transducer and a 4–8.5 MHz threedimensional transducer was performed for two‐dimensional and three-dimensional ultrasound.

### Genetic testing

Percutaneous cord blood sampling was performed on the foetus after informed consent was obtained. After termination of pregnancy, placental biopsies from the maternal and foetal sides were taken for subsequent analysis. Genomic DNA from the cord blood and the placental biopsies were prepared with centrifuge column. QF-PCR, CMA and Sanger sequencing were performed for foetuses to identified the cause of foetal abnormalities while the CNV-Seq and FISH were performed for placenta to identified the mosaicism ratio of trisomy 21. The following FISH probes (Abbott Molecular Inc., USA) specific to chromosome 13 (RB1 locus) which was as the control probe in our experiments and to three loci on chromosome 21(D21S259/D21S341/D21S342) were used to confirm abnormalities detected by CNV-seq. FISH was performed as recommended (Vysis, Inc.). One hundred nuclei were scored for detection of mosaicism. Primers used for QF-PCR were synthesized and labeled by Takara Biotechnology (Dalian) Co., Ltd. The short tandem repeat (STR) loci for aneuploid analysis located only in the 18, 13, 21 and sex chromosomes. CNV-Seq was performed by Semiconductor sequencing technique on the Bioelectronseq 4000 sequencing platform (CFDA registration permit NO. 20153400309), with a depth of 0.1 × . CMA was performed using a CytoScan 750 K array (Affymetrix, Santa Clara, CA, USA) according to the manufacturer’s recommendations. The data were analyzed by using Chromosome Analysis Suite v2.1 (Affymetrix) and Nexus Copy Number v.7.5 software (BioDiscovery, El Segundo, CA, USA). The entire coding sequence and the flanking intronic regions of *GATA1* were screened. Primers for amplification and sequencing were designed by using the University of California, Santa Cruz (UCSC) genomic browser (http://genome.ucsc.edu/) (Additional file 1: Table 1).

ALL of the CNVs and SNVs were identified based on associated records of the human reference genome 37 (NCBI37/hg19) of the National Centre for Biotechnology Information. Data were analysed in accordance with American College of Medical Genetics guidelines.

## Discussion

The incidence of MDS and AML is significantly increased in children with Down syndrome, and the World Health Organization (WHO) Classification of Tumours of Heamatopoietic and Lymphoid Tissues, revised 4^th^, and the recently released 5th edition of the WHO Classification and the 2022 International Consensus Classification of Myeloid Neoplasms and Acute Leukemia, classify both MDS and AML in children with Down syndrome as ML-DS [12–14]. ML-DS is usually preceded by a myeloproliferative neoplastic disease called TAM, which destroys megakaryocytic and erythroid differentiation. TAM is a pre-leukemic condition characterized by increased leukocyte counts and thrombocytopenia, as well as the presence of blasts in peripheral blood. The diagnosis of TAM requires a *GATA1* mutation as well as an increase in blasts and/or certain clinical features (especially hepatosplenomegaly) in neonate with Down syndrome and may also be mosaic [14]. The majority of cases of TAM appear in the first few days of life, but a small number may also appear during the foetal period, presenting as foetal hydrops or with features similar to those seen in the postnatal period [11]. In this paper, case 1 presented with foetal hepatosplenomegaly and case 2 with foetal hydrops.

A definitive diagnosis of TAM was made on the basis of various hematological and antenatal ultrasound presentations of the two foetuses, coupled with molecular testing demonstrating a mutation in the *GATA1* gene [15]. An increasing number of studies have shown that the *GATA1* gene is critical in the development of combined TAM and/or ML-DS in patients with trisomy 21. Jeffrey, Sylvia et al. found that *GATA1* mutations occur in utero, first appearing at 21 weeks of gestation [16–18], and will be undetectable when TAM is resolved [19]. GATA-binding Factor 1 (*GATA1*) is located on the X chromosome and is involved in the transcriptional regulation of erythrocyte-megakaryocytes [20, 21]. Back in 2002, John Crispino’s lab first identified an important role for *GATA1* mutations in ML-DS [22]. And nowadays, more than 100 types of *GATA1* mutations have been reported in trisomy 21 [23].

Most *GATA1* mutations in TAM include either frameshift or nonsense mutations within exon 2, the *GATA1* mutations create an early stop codon, resulting in a short isoform of the GATA1 protein that lacks the N-terminal activation domain, which then affect the translation of the GATA1 protein [1, 23]. The detection of *GATA1* mutations is important for the diagnosis of TAM. The hematopoietic transcription factor gene *GATA1* (localised at Xp11.23) is required for the development of megakaryocytes, erythrocytes, mast cells and eosinophils, and the dominance of the *GATA1* gene in combination with the gene dosage of the effect of trisomy 21 induces excessive proliferation of erythro-megakaryocytic blast cells [24]. Mutations in the *GATA1* gene are thought to be a pathognomonic feature of all myeloproliferative disorders in children with Down syndrome, including those with TAM [25]. Our study also suggests that the cause of TAM in foetuses may be due to mutations in the *GATA1* gene.

Genetic testing revealed that patient in case 1 carrying *GATA1* variant c.220G > A predicted a p. Val74Ile while the patient in case 2 carrying c.49dupC predicted a p. Gln17ProfsTer23. We evaluated the gene-disease association following the ClinGen Gene-Disease Validity Standard Operating Procedures, and curated the *GATA1* gene to “Definitive” grade associated with GATA1-Related X-Linked Cytopenia. Both of the variants are absent in the general population according to public databases (gnomAD, 1000 Genomes Project, and Exome Aggregation Consortium). Mutation c.220G > A has been reported in several research as pathogenic according to the ACMG guideline [26–28]. At the same amino acid c.220G > C (p.Val74Leu) change has been previously reported as pathogenic in the ClinVar database. The c.220G > A mutation is a substitution of a base at the 3′ end of the second exon of the *GATA1* gene. This mutation causes an aberrant splicing in the transcript, resulting in the production of a transcript that does not have the sequence from the second exon. Translation of this alternative transcript is initiated from the methionine in the third exon, resulting in the expression of the GATA1s protein. The variant c.49dupC has been reported by (PMID: 29,217,785) Séverine Drunat in a Down syndrome patient with like acute megakaryoblastic leukemia. Other variants at the same amino acid such as c.49_50del (p.Gln17fs) and c.49C > T (p.Gln17Ter) already provide to be pathogenic in the ClinVar database. Cabelof, DC et al. [29] and Kanezaki et al. [30] reported c.49 C > T in their case 10 and case 5, respectively. The consequence of the c.49dupC variation was similar to the base c.49 C > T substitution, which changed the amino acid sequence Gln17Ter. This mutation was a nonsense mutation resulting in a stop codon before Met84. Both of the mutations in our study, which located at the exon 2, highly possible to be disrupted the function of the protein and allow the abundant generation of truncated GATA1.

There is an interesting phenomenon in case 1, where we have demonstrated the presence of a low percentage of trisomy 21 mosaicism in the placenta. This result confirms that chromosomal inconsistencies between the placenta and the foetus are responsible for the false negative NIPT. On the other hand, there is also the possibility that contamination of placental tissue with circulating blasts carrying clonal trisomy 21 and GATA 1 mutations could lead to placental mosaicism with low proportions of trisomy 21. As mentioned in a previous case report by Roseman et al., it is difficult to determine whether T21 arose from an isolated de novo event or from low-level constitutional mosaicism [31]. After the foetus in Case1 was delivered, the foetal face did not show any obvious facial characteristics of Down syndrome, other than a collapsed nose. Therefore, we suspected that this might be a case of trisomy 21 mosiacism. This is because the cord blood chromosome results represent only the mesoderm of the embryo and are not representative of the other germ layers. In addition the foetus does not show any other manifestations of T21 other than TAM. However, we have been unable to confirm this hypothesis and we were unable to obtain the consent of the mother of the foetus to continue the study.

In conclusion, we identified two rare prenatal cases of foetuses with trisomy 21 combined with TAM. The [NM_002049.4 c.220G > A (p. Val74Ile) (Case 1)] mutation and the [NM_002049.4 c.49dupC (p. Gln17ProfsTer23) (Case 2)] mutation of *GATA1* were present, respectively. Further confirming the prenatal origin of the *GATA1* mutation. The presence of foetal trisomy 21 in the presence of prenatal ultrasound findings such as foetal hydrops or hepatosplenomegaly should be considered.

### Supplementary Information

**Additional file 1: Fig. 1.** Results of CNV-seq of the placenta. **A** CNV-seq results of biopsies from the centroid of the maternal surface of the placenta (Case 1): The calculated Z-score for trisomy 21 is 7.29. The proportion of trisomy 21 mosaicism is presumed to be 7% to 8%. **B** CNV-seq results of biopsies from the centroid of the foetal surface of the placenta (Case 1): The calculated Z-score for trisomy 21 is 6.75. Trisomy 21 with a mosaicism rate of 6% to 7%. **C** Normal result for chromosome 21(as a comparison).

**Additional file 2: Fig. 2.** Interphase fluorescent in situ hybridization analysis of uncultured placental cells using the commercial DNA probes (Abbott Molecular Inc., USA), chromosome 13 (RB1 locus)which was as the control probe in our experiments, and three loci on chromosome 21(D21S259/D21S341/D21S342) were used to confirm abnormalities detected by CNV-seq. (RB1, spectrum green) and (D21S259/D21S341/D21S342, spectrum red) shows(A) a trisomy 21 cell with three red signals and two green signals, and(B) a normal disomy 21 cell with two red signals and two green signals.

**Additional file 3: Table 1.** List of primers designed using UCSC genomic browser tool to amplify the exonic regions of GATA1 gene.

## Data Availability

Not applicable.
