# Supplementary figures and images for: Prenatal diagnosis of Down syndrome combined with transient abnormal myelopoiesis in foetuses with a GATA1 gene variant: two case reports
Source: Mol Cytogenet. 2023 Oct 19;16:27. doi: 10.1186/s13039-023-00658-w (PMC10588144; doi:10.1186/s13039-023-00658-w)

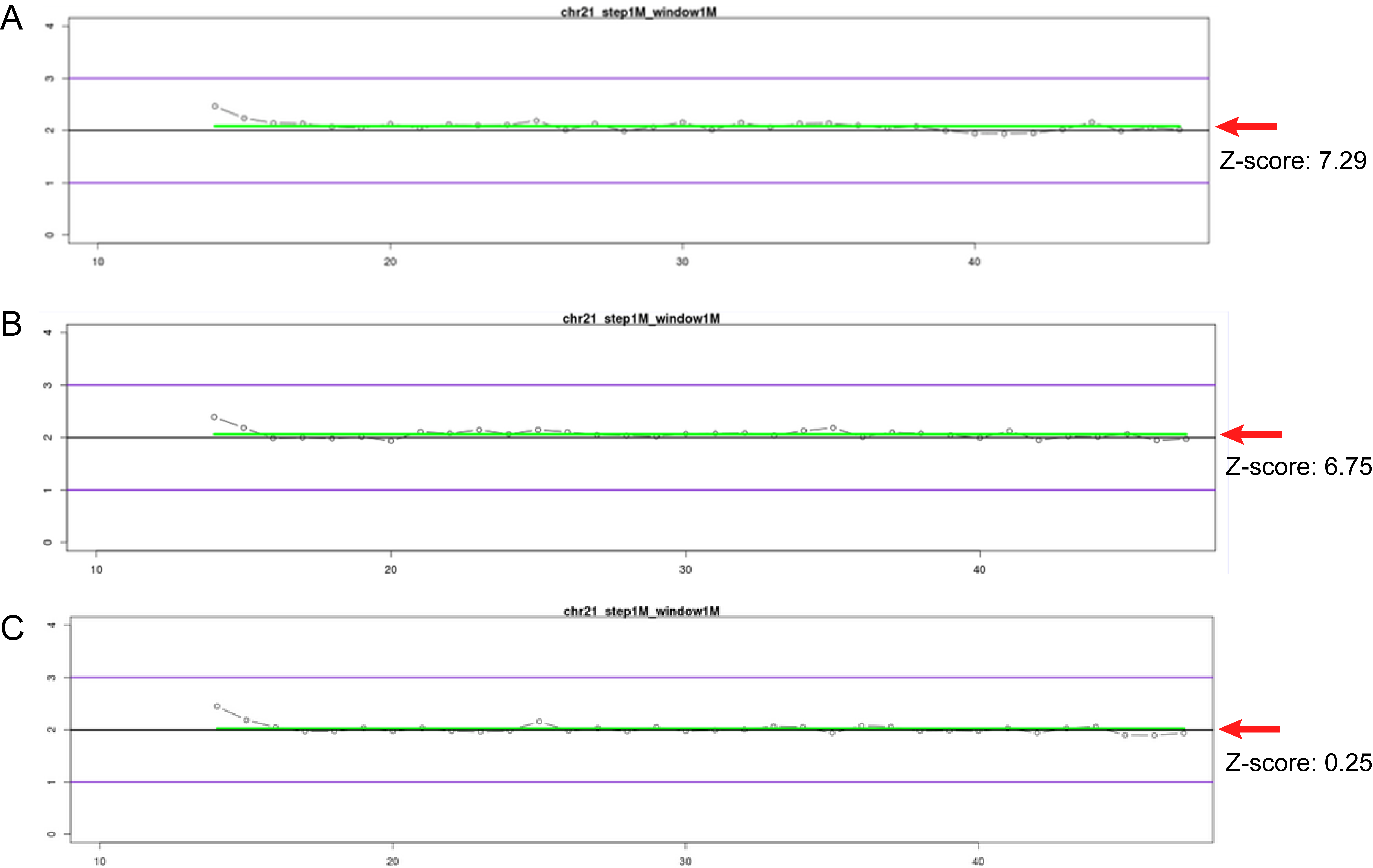

Supplement: Supplementary file 1 — Additional file 1: Fig. 1. Results of CNV-seq of the placenta. A CNV-seq results of biopsies from the centroid of the maternal surface of the placenta (Case 1): The calculated Z-score for trisomy 21 is 7.29. The proportion of trisomy 21 mosaicism is presumed to be 7% to 8%. B CNV-seq results of biopsies from the centroid of the foetal surface of the placenta (Case 1): The calculated Z-score for trisomy 21 is 6.75. Trisomy 21 with a mosaicism rate of 6% to 7%. C Normal result for chromosome 21(as a comparison). [file 13039_2023_658_MOESM1_ESM.tif]

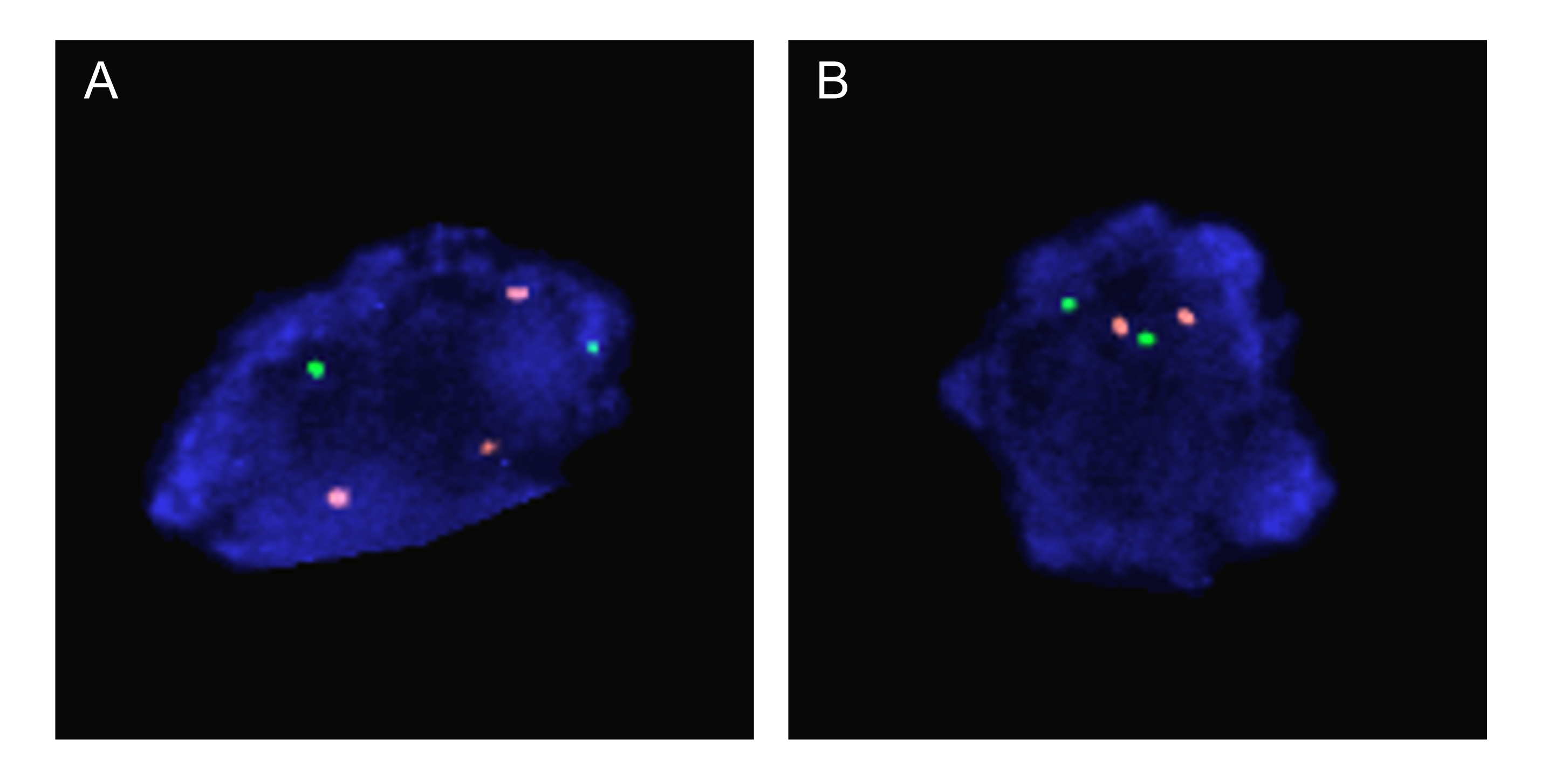

Supplement: Supplementary file 2 — Additional file 2: Fig. 2. Interphase fluorescent in situ hybridization analysis of uncultured placental cells using the commercial DNA probes (Abbott Molecular Inc., USA), chromosome 13 (RB1 locus)which was as the control probe in our experiments, and three loci on chromosome 21(D21S259/D21S341/D21S342) were used to confirm abnormalities detected by CNV-seq. (RB1, spectrum green) and (D21S259/D21S341/D21S342, spectrum red) shows(A) a trisomy 21 cell with three red signals and two green signals, and(B) a normal disomy 21 cell with two red signals and two green signals. [file 13039_2023_658_MOESM2_ESM.tif]
