# Supplementary material for: Prenatal diagnosis of Down syndrome combined with transient abnormal myelopoiesis in foetuses with a GATA1 gene variant: two case reports
Source: Mol Cytogenet. 2023 Oct 19;16:27. doi: 10.1186/s13039-023-00658-w (PMC10588144; doi:10.1186/s13039-023-00658-w)
Supplement: Supplementary file 3 — Additional file 3: Table 1. List of primers designed using UCSC genomic browser tool to amplify the exonic regions of GATA1 gene. [file 13039_2023_658_MOESM3_ESM.docx]

**Supplementary Table 1.**

List of primers designed using UCSC genomic browser tool to amplify the exonic regions of GATA1 gene.

| Primer set | Primer sequences | | Amplified segments |
| --- | --- | --- | --- |
|  | Forward | Reverse |  |
| 1 | FP-5′-GGAAAGGAGGAAGAGGAGC -3′ | RP-5’- GAGAAGCTTCCAGCCATTTC -3′ | GATA1-  Exon2 |
| 2 | FP-5′- GAACCACTGCACCCTGAC -3′ | RP 5′- GAAGAGGGAGCTAGGCTCAG -3′ | GATA1-  Exon3 |
| 3 | FP-5′- GACAGGGAAGTTGAGGTGG -3′ | RP 5′- TAATGGGAAGATGTGGCTTC -3′ | GATA1-  Exon4 |
| 4 | FP-5′- CTTGGGTCCTCCTGACATC -3′ | RP-5’- AACAGGAACAGAGTGGGG -3′ | GATA1-  Exon5 |
| 5 | FP-5′- AGTGGGGTAGAGAGGGTGTC -3′ | RP-5’- CAGAGACTTGGGTTGTCCAG -3′ | GATA1-  Exon6 |

Conditions used to perform the polymerase chain reaction to amplify the target region of GATA1.

| Tm  ( °C) | PCR conditions | | | | |
| --- | --- | --- | --- | --- | --- |
|  | 95°C  use | Thermal cycle (40 cycles) | | | 25°C  second |
|  |  | 95°C  (seconds) | Tm (seconds) | 72°C  (seconds) |  |
| 58 | 5 | 30 | 45 | 50 | Hold |
